# *Lactiplantibacillus plantarum*–Nomad and Ideal Probiotic

**DOI:** 10.3389/fmicb.2021.712236

**Published:** 2021-10-06

**Authors:** Mario Fidanza, Pinaki Panigrahi, Tobias R. Kollmann

**Affiliations:** ^1^Telethon Kids Institute, Subiaco, WA, Australia; ^2^Georgetown University Medical Center, Department of Pediatrics, Washington, DC, United States

**Keywords:** probiotics, microbiome, *Lactobacillus*, immunology, sepsis, infection, innate immunity

## Abstract

Probiotics are increasingly recognized as capable of positively modulating several aspects of human health. There are numerous attributes that make an ideal probiotic. *Lactiplantibacillus plantarum* (Lp) exhibits an ecological and metabolic flexibility that allows it to thrive in a variety of environments. The present review will highlight the genetic and functional characteristics of Lp that make it an ideal probiotic and summarizes the current knowledge about its potential application as a prophylactic or therapeutic intervention.

## *Lactobacillus Plantarum*–Nomad and Ideal Probiotic

Probiotics are defined as living organisms which, when administered in adequate amounts, confer a health benefit to the host (FAO/WHO 2006). While probiotics have been used in some capacity for centuries, the first report of their proposed health benefits was published more than 100 years ago by Elie Metchnikoff, suggesting that optimizing the composition of intestinal microbes would have a positive effect on health (Metchnikoff, 1907). This has remained precisely what the application of probiotics attempts to accomplish.

Humans are colonized by a diverse and dynamic consortium of microbes that in total outnumber the cells that comprise the human body itself. This consortium is comprised of bacteria viruses, archaea, and other eukaryotic organisms (Cho and Blaser, 2012). The total collection of microbes inhabiting the human body is referred to as the microbiota. The microbiota interacts with the host in a symbiotic relationship and has been shown to play a major role in human immunology, metabolism, and also in multiple disease pathologies (Ogunrinola et al., 2020). Establishment of the microbiota begins before birth, i.e., *in utero* and undergoes rapid sequential compositional changes from the time of birth onward to about 2 years of age when stabilization occurs and the microbiota, compositionally, begins to resemble that of a healthy adult (Greenhalgh et al., 2016; Kapourchali and Cresci, 2020). Thus, early life is the key period during which the microbiota is established (Tamburini et al., 2016; Du Toit, 2017). Of note, this early life period is also characterized by the rapid maturation of immune, metabolic, and neurological pathways (Brook et al., 2017; Harbeson et al., 2018). Given this parallel high-speed development, it should come as no surprise that the microbiota plays a significant role in shaping the maturation and eventual outcomes of many of these developmental pathways (and possibly vice versa). Together, changes across this period exert a profound impact on human health for years, decades - if not life (Zeissig and Blumberg, 2014; Stiemsma and Michels, 2018).

There are several factors that influence early-life microbiota composition and development, such as gestational age at birth, mode of delivery, post-natal feeding practices and environmental factors such as antibiotic exposure (Guo et al., 2021). When the normal patterns of development or the composition of the microbiota are altered in such a way that unfavorable or pathogenic bacteria become more dominant, the microbiota assumes a state of dysbiosis. Dysbiosis has been implicated in a number of disease pathologies including allergy, type-1 diabetes, auto-immune disorders, and metabolic disorders such as obesity (Tilg and Kaser, 2011; Azad et al., 2015; Kostic et al., 2015). The broad overall objective of probiotics is to prevent or ameliorate these extreme states of dysbiosis in order to establish a balanced microbiota that imparts positive benefits to the host (McFarland, 2014; Gagliardi et al., 2018).

Given this complex, interwoven system the successful application of probiotics must take into account several aspects. One such consideration is the timing of administration. As the microbiota from the time of birth to ~2 years of age is characterized as being highly dynamic, it has been theorized that this period of life represents an intervention window during which probiotic administration can have a meaningful and lasting impact (Du Toit, 2017). Vertical transmission of microbes from mother to infant before and during delivery and subsequently through breastfeeding establishes a situation where interventions could be administered to either the infant and/or the mother in order to achieve positive health benefits (Asnicar et al., 2017; Renelies-Hamilton et al., 2021). There has been several studies assessing the impact of probiotic administration to the pregnant mother, the newborn infant or the nursing mother. Indeed, several recent reviews and meta-analyses of published data indicate probiotic supplementation during this critical window is associated with many positive health benefits (Gritz and Bhandari, 2015; Reid, 2016; Zhang et al., 2016; Baldassarre et al., 2018; Liu et al., 2018; Tsai et al., 2019; Underwood, 2019).

In pregnant mothers, bacterial vaginosis and preeclampsia have been definitively linked to pre-term birth which is associated with numerous short and long-term health complications for the infant. Meta-analyses of published studies suggest that the administration of probiotics to pregnant mothers can reduce the incidence of both bacterial vaginosis and preeclampsia (Lindsay et al., 2013; Huang et al., 2014). A direct link between probiotic supplementation and pre-term birth outcomes has been harder to establish (Othman et al., 2007; Jarde et al., 2018). However, a recent study demonstrated that administration of a probiotic mixture containing *S. faecalis, C. butyricum*, and *B. mesentericus* to high-risk pregnant women was associated with a lower rate of spontaneous pre-term birth, increased gestational age at delivery and decreased intrauterine infections compared to a no probiotic control group (Kirihara et al., 2018). Most studies assessing the impact of probiotic administration to the newborn have been conducted in premature or low birth weight infants. Premature infants are at an increased risk for several adverse outcomes; the impact of probiotic administration on clinically relevant outcomes can therefore be more readily determined. Meta-analyses of these studies have demonstrated that the administration of probiotics significantly reduces the risk of necrotizing enterocolitis, late-onset neonatal sepsis and atopic dermatitis (Zago et al., 2011; Zhao M. et al., 2018; Underwood, 2019).

Another consideration for the successful application of probiotics is the species, strain and composition of the probiotic product that is utilized. There are several functional properties that are required for a bacterial species to be an effective probiotic. Primary characteristics that must be considered are resistance to gastric acid and bile salts, ability to adhere to the intestinal mucosa, and finally sensitivity to antibiotics. Specifically, potential probiotics are evaluated for their ability to persist in the host, favorably alter intestinal microflora, promote intestinal integrity and mobility, modulate host immune responses and finally for their antimicrobial or competitive activity against potentially pathogenic bacteria.

Lactic acid bacteria (LAB) have long been appreciated for their probiotic properties. The health benefits associated with certain fermented foods, which are generally considered rich sources of LAB, have been recognized for millennia. More definitively, even the original Metchnicoff essay identified LAB as potentially being capable of offsetting the effects of harmful bacteria and conferring health benefits on the host. It is therefore not entirely surprising that *Lactobacillus* and *Bifidobacterium* are the most extensively studied bacterial genera in probiotic research. Within these genera, several species and strains with positive health benefits have been identified. Of these, *Lactiplantibacillus plantarum* (formerly *Lactobacillus plantarum)* is an interesting probiotic candidate. It has been demonstrated to possess several fundamental probiotic characteristics, is highly versatile and is capable of successfully colonizing and inhabiting the human gastrointestinal system. This review will cover the characteristics of *L. plantarum* that make it an ideal probiotic candidate as well as highlight the current evidence that administration of *L. plantarum* can confer positive health benefits to humans.

## Lactiplantibacillus Plantarum

*Lactiplantibacillus plantarum* is a gram-positive lactic acid bacteria species. *L. plantarum* exhibits ecological and metabolic adaptability and is capable of inhabiting a range of ecological niches including fermented foods, meats, plants, and the mammalian gastro-intestinal tract (Filannino et al., 2018). Often, the ability of different bacterial strains to adapt to defined environments is thought to be accomplished by genome specialization that facilitates niche-specific phenotypic fitness. This typically occurs through a process of genome decay in unutilized genes and enrichment of those that impart habitat-specific fitness. This adaptive process generally ensures that individual bacterial strains isolated from the same ecological niche cluster genetically and carry similar niche specific genetic signatures. This has been demonstrated to be the case for *L. gasseri* and *L. jensenii* isolated from human vaginal environments and *L. reuteri* strains isolated from different vertebrate intestinal tracts (Kleerebezem et al., 2003; Mendes-Soares et al., 2014). However, a recent analysis assessing dozens of *L. plantarum* strains for genomic signatures that reflect specific environmental adaptations, demonstrated that this is not the case in *L. plantarum*. Thus, the evolutionary history of this bacterial species does not appear to be directly related to the features of the niches from which they were isolated. This suggests that, unlike most *Lactobacillus* species, *L. plantarum* may acquire and preserve functional characteristics that are not solely determined by a single habitat (Martino et al., 2016; Filannino et al., 2018). In this manner, *L. plantarum* maintains a diverse functional genome that facilitates a metabolic flexibility that allows it to colonize a variety of environments (Martino et al., 2016; Filannino et al., 2018; Inglin et al., 2018).

## Resistance to the Conditions of the Human Gastrointestinal Tract

A major hurdle for any probiotic is surviving the harsh conditions of the human gastrointestinal tract. To be effective, a probiotic must first be able to withstand the acidic conditions of the stomach, and then tolerate exposure to bile acids in the small intestine. The stomach is an inhospitable environment. Human gastric juice is primarily composed of pepsin and hydrocholoric acid. This composition results in a cycling pH in the stomach that ranges from about 1.5 during fasting to between 3 and 5 after food intake (Schubert, 1999). Acid resistance is thus considered a primary criterion for the selection of probiotic bacterial strains.

*Lactobacillus* species employ several strategies to facilitate acid tolerance. These include mechanisms to maintain intracellular pH homeostasis, rapid recycling of damaged proteins and induction of various stress response pathways (Chiang et al., 2014; Wu et al., 2014). Additionally, the acid tolerance of several *Lactobacillus* strains, including *L. plantarum* can be increased by previous exposure to non-lethal acidic conditions in a process known as the acid tolerance response (Foster and Hall, 1991). It is well-established that different strains of the same *Lactobacillus* species have highly variable acid tolerance. While this is also true for *L. plantarum*, numerous studies have demonstrated that the majority of tested *L. plantarum* strains exhibit acid tolerance, albeit to slightly different degrees, through a number of specific and distinct mechanisms (Kaushik et al., 2009; Guidone et al., 2013; Hamon et al., 2014).

Specifically, the acid tolerance mechanisms employed by *L. plantarum* include inducible alterations to the fatty acid composition of the plasma membrane such that the ratio of saturated to unsaturated fatty acids increases upon exposure to low pH conditions causing a significant reduction in membrane fluidity (Huang et al., 2016). Additionally, *L. plantarum* has also been shown to upregulate the expression of phosphofructokinase (*pfk)* and pyruvate-kinase (*pyk)* to facilitate higher rates of sugar metabolism, and in result, greater ATP production which provides energy to drive proton pumps that help maintain intracellular pH homeostasis (Huang et al., 2016). This increase in proton pump function is further facilitated by increased expression of ATP-synthase genes *atpA* and *atpC* (Heunis et al., 2014; Seme et al., 2015; Huang et al., 2016). Finally, *L. plantarum* has also been shown to alter its amino acid metabolism upon exposure to acidic environments. Amino acid metabolism has been shown to play a significant role in maintaining cellular homeostasis and promoting resistance to environmental stresses in multiple lactic acid bacteria species (Reveron et al., 2012; Heunis et al., 2014; Seme et al., 2015). It has been demonstrated that the intracellular concentration of alanine and arginine in particular increase in *L. plantarum* upon exposure to acid stress in a metabolic response that promoted low pH tolerance.

After enduring the low pH of the stomach, a probiotic must also be able to tolerate exposure to bile salts in the small intestine. Bile salts are synthesized in the liver from cholesterol that is conjugated to glycine or taurine residues. These compounds are then released into the small intestine and have a primary role in lipid metabolism. Through detergent disruption of cellular membranes, DNA damage and oxidative stress, bile salts pose a significant challenge for probiotic survival. Bile tolerance is thus another primary criterion for the selection of potential probiotics. While *L. plantarum* employs multiple strategies to withstand the stress of bile salt exposure, there are 4 that are considered critical: (i) induction of bile salt hydrolases (ii) altering membrane composition and fluidity (iii) protecting against oxidative injury and finally (iv) maintaining the proton motive force.

Bile salt hydrolase (*bsh)* is generally considered a major component of bacterial bile tolerance. It is responsible for catalyzing the de-conjugation of glycine and taurine residues from cholesterol. While the exact nature of how bile salt de-conjugation limits the negative impact on cellular homeostasis is unclear, *bsh* expression has been definitively linked to bile tolerance in a number of *Lactobacillus* species (Begley et al., 2006; Pfeiler et al., 2007; Pfeiler and Klaenhammer, 2009). *L. plantarum* carries four *bsh* genes that are upregulated upon exposure to bile salts (Bron et al., 2006; Duary et al., 2012; Gu et al., 2014). The products of these genes represent the first line of bacterial defense against the destructive effects of bile salts in the small intestine.

The commonly characterized *L. plantarum* WCSF1 demonstrates very good tolerance to bile salts in an *in vitro* artificial GI tract environment; this tolerance was accompanied by significant changes in cell morphology (Bron et al., 2004). The morphological change facilitating this tolerance was at least partially attributed to the upregulation of *Cfa2*, a cyclopropane-fatty-acyl-phospholipid synthase that has been shown to alter cell membrane composition and fluidity perhaps imparting resistance to bile salt-induced detergent disruption of cellular membranes (Hamon et al., 2011). To protect against oxidative insult, *L. plantarum* strains have been shown to upregulate the stress-induced glutathione reductase gene and the *metC-cysK* operon upon exposure to bile stress. Finally, by upregulating expression of the F0F1-ATPase, *L. plantarum* facilitates the expulsion of protons from the intracellular environment to help maintain pH homeostasis when exposed to alkaline bile salts (Bron et al., 2004, 2006; Hamon et al., 2011).

## Adherence to Intestinal Mucosa

To impart positive health benefits, a probiotic must be able to colonize and persist in the human gastrointestinal tract. Therefore, an important probiotic selection criterion is the ability to adhere to the human intestinal mucosa. The mucus layer coating the epithelial cells of the gastrointestinal tract serves as a physical barrier to protect against the invasion of pathogenic microbes; on the other hand, it also serves as an important colonization site for commensal bacteria. The intestinal mucus is comprised primarily of mucins and glycoproteins that assemble into a complex net-like structure that attaches to epithelial cells (Sicard et al., 2017). While the thickness of this mucus layer is variable throughout the digestive tract, in the colon, where microbe concentration is highest, the innermost and densest portion of the mucus layer is typically devoid of bacteria in healthy individuals. Bacterial persistence is therefore largely related to the ability to adhere to mucus or extra-cellular matrix (ECM) components rather than epithelial cells themselves which are typically only in direct contact with invasive pathogenic microbes in the context of certain disease pathologies.

It has been demonstrated that while trypsin treatment significantly reduced the adhesion capacity of *L. plantarum*, it did not eliminate adhesion entirely (Wang G. et al., 2018). This is consistent with other reports on the adhesion properties of *Lactobacillus* species and suggests that non-protein interactions contribute to the adhesion of *L. plantarum* (Deepika and Charalampopoulos, 2010; Jensen et al., 2014). In fact, the initial adhesion interaction is believed to involve non-specific mechanisms. Physical characteristics of the bacterial cell such as surface charge, hydrophobicity and electron donor-acceptor properties can influence bacterial adhesion properties. While the adhesion capacity of *L. plantarum* has been demonstrated to be strain dependent, a positive correlation was found between cell surface hydrophobicity and adhesion capacity (Yadav et al., 2013; Buntin et al., 2017).

In terms of specific interactions, a number of prototypical cell-surface bacterial adhesins contribute to the adhesion capacity of *L. plantarum*. Mannose-specific adhesion (*Msa)*, has been identified as a “probiotic gene” and has been definitively linked to mannose-binding and adhesion capacity in *L. plantarum* (Gross et al., 2010; Buntin et al., 2017). As a result, inter-strain variation in the *Msa* gene, particularly in relation to the number of PxxP-repeats in the mucus-binding (MUB) domain, has been associated with inter-strain differences in the adhesive characteristics of *L. plantarum* (Gross et al., 2010). A number of additional adhesin proteins have been implicated in the capacity of *L. plantarum* to adhere to components of the intestinal mucosa. Mucus-binding protein (MucBP) and mucus-adhesion binding protein (MapA) have been shown to be central in the mucin binding capacity of *L. plantarum*. Finally, collagen binding protein (Cbp) and alfa-enolase-1 (EnoA1), a fibronectin binding protein facilitate the binding of *L. plantarum* to epithelial ECM component in *in vitro* models (Ramiah et al., 2007; Castaldo et al., 2009; Gross et al., 2010; Yadav et al., 2013; Buntin et al., 2017).

Recently, a number of unlikely proteins have also been shown to contribute to bacterial adhesion to the intestinal mucosa. These proteins have been termed “moonlighting proteins” in light of their function in several distinct cellular processes. These proteins are generally well-conserved across bacterial species, and often have central roles in key cellular functions such as metabolism and stress responses (Wang et al., 2014). While the mechanisms have not been completely resolved, two moonlighting proteins have been specifically associated with the adhesion capacity of *L. plantarum*. Elongation-factor-Tu (EF-Tu) (Dhanani and Bagchi, 2013) and glyceraldehyde-3-phosphate dehydrogenase (GAPDH) (Wang G. et al., 2018) have been demonstrated to contribute to the adhesion of *L. plantarum*. Antibodies targeting these surface proteins significantly reduced the ability of *L. plantarum* to adhere to both the HT-29 epithelial cell line and porcine mucin. Interestingly, addition of purified EF-Tu or GAPDH significantly increased the adhesion of strains that previously had poor adhesion ability suggesting that these proteins are capable of self-assembly and integration to facilitate bacterial adhesion. Further work will be required to uncover the mechanism underlying how these and other moonlighting proteins contribute to bacterial adhesion.

Most assessments of bacterial adhesion are conducted *in vitro*. These studies generally evaluate the adherence of bacterial strains to immobilized mucin (usually porcine), extra-cellular matrix components or epithelial cell cultures, which generally utilize the HT-29 or Caco-2 lines. Animal models can be used to further validate these *in vitro* findings. Administration of *L. plantarum* has been shown to achieve moderate or long-term colonization of the gastrointestinal tract of both mice and rats (Wang et al., 2009; Daniel et al., 2013). While these studies are informative, they fail to capture the complexities of adherence to the human intestinal mucosa. Human mucosal tissue is obviously more difficult to obtain, therefore probiotic persistence is most commonly evaluated by fecal recovery, that is the duration of time over which the probiotic can be found in fecal samples of subjects who had been administered the probiotic. The number of studies assessing *L. plantarum* persistence in humans is relatively limited. However, Panigrahi and colleagues demonstrated that 7-day administration of *L. plantarum* in combination with a fructooligosaccharide prebiotic to healthy newborns for 7 days resulted in long-term colonization, with a significant number of infants (32%) remaining colonized 6 months after treatment cessation (Panigrahi et al., 2008). These *in vivo* results further validate *in vitro* assessments and suggest that *L. plantarum* is capable of adhering to the human intestinal mucosa and persisting in the mammalian gastrointestinal tract.

## Promotion of Intestinal Integrity or Motility

One mechanism by which probiotics may positively influence human health is through the promotion of intestinal integrity or motility. The intestinal epithelial layer is the major barrier that separates the body from the external environment and the consortium of microbes that colonize the gastrointestinal tract. The intestinal epithelial monolayer is comprised of different specialized epithelial cells such as enterocytes, Paneth cells and goblet cells, each of which has distinct functions (Chelakkot et al., 2018). The most abundant of these are enterocytes or intestinal epithelial cells. The primary function of enterocytes is maintenance of barrier integrity, which entails permitting the passage of water and nutrients but preventing the transit of pathogenic microbes or their associated toxins (Chelakkot et al., 2018). Epithelial tight-junction proteins equip the intestinal epithelial cells with this functional capacity by sealing the intercellular space between cells and restricting the transport of molecules across the epithelial barrier. These tight junctions are composed of several transmembrane and cytosolic proteins that cooperate to efficiently adapt to different environmental demands by sealing or opening to maintain paracellular transport under different physiological conditions. Disruptions to tight junction assembly of function, result in changes in epithelial permeability and can have serious effects on human health. For example, decreased expression of known tight junction proteins such as ZO-1 and occludin, have been shown to be associated with pathologies such as necrotizing enterocolitis (NEC) and inflammatory bowel disease (Hogberg et al., 2013).

*L. plantarum* has been shown to modulate epithelial barrier function both *in vitro* and *in vivo* through a number of mechanisms. Wang and colleagues demonstrated that administration of *L. plantarum* to piglets significantly counteracted or prevented increases in gut permeability induced by enterotoxigenic *Escherichia coli* (Wang J. et al., 2018). This protective benefit was shown to be conferred through multiple overlapping mechanisms that included maintaining expression of critical tight-function proteins, reducing expression of pro-inflammatory cytokines and modulating gut microbiota composition. This finding is further corroborated by a study in a rat model of NEC in which administration of *L. plantarum* significantly increased the expression of ZO-1 and improved intestinal barrier integrity (Blackwood et al., 2017). However, this beneficial outcome was only observed when *L. plantarum* was administered in combination with the likely pathogenic *Cronobacter sakazakii*. The integrity-protective responses induced by *L. plantarum* may be at least partially mediated through the engagement and activation of TLR-2 in the intestinal epithelium. Specifically, in a trial assessing the effect of *L. plantarum* administration on tight-junction physiology in healthy humans, Karczewski and colleagues demonstrated that *L. plantarum* induced increased expression and trafficking of ZO-1 and occludin tight junction sites through a mechanism that was mediated by TLR-2 signaling (Karczewski et al., 2010).

A recent study also highlighted a role for the *L. plantarum* cell surface protein known as micro integral membrane protein (MIMP) in improving intestinal barrier integrity in a murine DSS induced IBD model (Yin et al., 2018). By modulating lipopolysaccharide (LPS) responses, *L. plantarum* derived MIMP was shown to significantly reduce the production of inflammatory cytokines and in turn significantly reduce gut permeability and the associated IBD pathology (Chelakkot et al., 2018). While more evidence is needed, particularly in *in vivo* models, this evidence suggests that *L. plantarum* is capable of impacting intestinal barrier integrity, particularly when underlying pathologies are present.

Promoting intestinal motility is another possible mechanism through which probiotics can positively influence host health. While specific probiotic strains such as *Bifidobacterium lactis* have been definitively linked to improving intestinal motility, modulating gastrointestinal transit time and alleviating the symptoms associated with constipation, similar studies regarding *L. plantarum* are relatively sparse. Recently however, it has been demonstrated that administration of *L. plantarum* was able to prevent or significantly reduce the symptoms of charcoal induced constipation in mice (Zhao X. et al., 2018). Importantly, a randomized double-blind clinical trial in constipated adults in Malaysia demonstrated that a combination synbiotic preparation of *L. plantarum, B. lactis*, and inulin-oligofructose once daily for 12 weeks significantly improved defecation frequency and stool type score (Bristol Stool Form Scale) (Lim et al., 2018). The direct influence of *L. plantarum* in this context is difficult to assess since it was used in combination with *B. lactis* which has previously demonstrated to alleviate symptoms of constipation when administered as a stand-alone probiotic.

## Ability to Alter Intestinal Flora and Inhibit the Growth of Potential Pathogens

The capacity of LAB species to inhibit the growth of pathogenic bacteria has been recognized for centuries, hence the role they have played in preventing food spoilage. The ability to alter the composition of the intestinal flora and inhibit the colonization or growth of pathogenic microbes is a hallmark characteristic of probiotics. There are several mechanisms through which this biological activity can be accomplished, the principal of which include: competition for nutrients and adhesion sites, inducing changes in environmental conditions that are unfavorable to pathogenic bacteria, production of antimicrobial compounds and finally by modulating host immune responses (Corr et al., 2009; Hu et al., 2017).

Unraveling the individual contribution of each of these mechanisms to the overall capacity of specific probiotics to impact the growth of other microbes is remarkably difficult. In reality, these mechanisms likely act concurrently and cooperatively to influence the composition of the host microbiota and prevent the colonization or persistence of pathogenic bacteria. Comparative studies assessing the antimicrobial activity of various probiotic strains have demonstrated that *L. plantarum* frequently exhibits the broadest capacity to inhibit pathogen growth amongst *Lactobacilli* species (Davoodabadi et al., 2015; Ren et al., 2017; Ahn et al., 2018; Arena et al., 2018; Kim A. R. et al., 2019).

Antimicrobial substances produced by probiotics are often naturally occurring bacterial metabolites and can generally be classified into low and high molecular mass compounds (<1,000 and >1,000 Da). The low-molecular mass group, also referred to as the non-bacteriocin group, includes compounds from a wide range of chemical classes such as organic acids, hydrogen peroxide, acetyl-aldehyde, and carbon dioxide. The high-molecular mass group is comprised primarily of proteinaceous compounds referred to as bacteriocins (Arena et al., 2018). Bacteriocins are ribosomally synthesized peptides with antibacterial activity (Cavera et al., 2015; Dicks et al., 2018). Though classification practices are evolving, four classes have been proposed to characterize LAB derived bacteriocins. Class I bacteriocins are referred to as lantibiotics and are characterized primarily based on their post-translational modification. Class II bacteriocins are small, heat-stable non-lanthionine containing bacteriocins that can be sub-divided into 4 distinct subclasses based on their molecular characteristics. Class III bacteriocins are larger peptides of over 30 kDa that often act in protein-like complexes (Mokoena, 2017). Finally, Class 4 bacteriocins are large complexes that contain lipid or carbohydrate moieties (Wang Y. et al., 2018). The variety of bacteriocins produced by *L. plantarum* generally belong to Class II and are collectively referred to as plantaricins. Plantaricins may be either chromosomally or plasmid encoded and are usually organized within operon clusters that consist of a structural gene(s), an immunity gene, an ABC transporter and an accessory protein that facilitates export (Pal and Srivastava, 2015).

A number of plantaricin compounds have been identified. Broadly, the mechanism of action of these plantaricin compounds is disruption and/or perforation of target cell membranes. In regard to pathogenic bacteria, *L. plantarum* derived plantaricin compounds have shown to be exceptionally effective against gram-positive bacterial pathogens such as *Micrococcus luteum* (Jiang et al., 2018), *Listeria monocytogenes* (Chen et al., 2018), and *Staphylococcus aureus* (Song et al., 2014). Traditionally, plantaricin compounds have been thought to be less effective against gram-negative bacteria, likely due to the outer-membrane of these bacteria providing a protective barrier against plantaricin induced disruption or perforation of the cytoplasmic membrane (Pal and Srivastava, 2014). However, some specific plantaricin compounds such as plantaracin E/F and J/K dipeptides can effectively inhibit gram-negative bacteria like *Escherichia coli* in specific circumstances (Pal and Srivastava, 2014; Song et al., 2014). Interestingly, while also effective on their own, combinations of plantaricin EF and JK potentiated the effect of traditional antibiotics against *Staphylococcus epidermis*, including re-sensitizing the clinically derived antibiotic resistant *S. epidermis 126* strain to gentamicin and tetracycline (Selegard et al., 2019). Select plantaricin species have also shown activity against viruses such as Influenza (Park et al., 2013; Jiang et al., 2017) and fungal species such as *Candida albicans* (Sharma and Srivastava, 2014), *Aspergillus parasiticus* and *Penicillium expansum* (Luz et al., 2018).

In addition to the production and release of antimicrobial compounds, probiotics can inhibit the growth or colonization with potential pathogens by competing for nutrients or adhesion sites. The antagonistic effects of probiotic strains on the adhesion of pathogenic microbes are generally evaluated using human colonic cell lines such as Caco-2 *in vitro* and often also incorporate variables such as low pH and bile-salt exposure in order to better simulate conditions of the human gastrointestinal tract. Lau and Chye demonstrated that *L. plantarum* 0612 was able to competitively inhibit and even displace both *E. coli* and *L. monocytogenes* from Caco-2 monolayers (Chye, 2018). Interestingly, this antagonistic potential was actually increased after *L. plantarum* was exposed to gastrointestinal transit simulation. As the ability to inhibit the adhesion of pathogens is, like many probiotic traits, strain specific, it is also important to note that *L. plantarum* strains derived from humans have been demonstrated to be capable of inhibiting the adhesion of a number of pathogenic microbes to Caco-2 cells by competition and displacement (Jiang et al., 2016).

The ability of *L. plantarum* to inhibit the growth or adhesion of pathogenic microbes has been well-studied *in vitro*, however, because assessing similar characteristics *in vivo* is more complicated, such evidence is substantially more limited. Treatment of C57BL/6J mice with *L. plantarum* has been shown to prevent gastric inflammation and accompanying changes in gastric microbiota composition that are induced by *Helicobacter pylori* infection and DSS treatment (Pan et al., 2016; Zhang et al., 2019). Similarly, mixed *L. plantarum* strains administered to mice prior to or as treatment for *Staphylococcus aureus* infection significantly reduced intestinal inflammation, increased sIgA levels and prevented *S. aureus* induced pathogenic shifts in intestinal microbiota composition (Ren et al., 2017). Finally, in both high-fat diet mouse and rat obesity models, administration of *L. plantarum* alone or in combination with *L. fermentum* not only reduced fat retention but caused a dramatic shift in intestinal microbiota composition via enrichment of *Bifidobacterium* and *Lactobacillus* species relative to controls (Xiuliang Li Xiuyan et al., 2018; Hussain et al., 2020).

In the absence of metabolic disruption or pathogenic challenge, the capacity of *L. plantarum* ZDY2013 to positively influence the composition of the microbiota in healthy mice has been assessed in two independent studies. Using qPCR, Huang and colleagues demonstrated that 2-week administration of *L. plantarum* enhanced the populations of *Bifidobacterium* and *Lactobacillus* species in the colon and cecum and reduced the abundance of potentially enteropathogenic *Enterococcus* and *Clostridium* species (Huang et al., 2015). A separate and more detailed analysis using 16s rDNA sequencing also demonstrated that short and long-term administration of *L. plantarum* significantly altered the microbial composition of the small-intestine of C57BL/6J mice. Although transient, administration of *L. plantarum* led to a significant increase in the richness and diversity of the microbial composition of the small intestine (Xie et al., 2016; Linninge et al., 2019). This change was accompanied by enrichment of numerous *Proteobacteria* species and a corresponding decrease in the abundance of *Bacteriodetes*. While these compositional changes only persisted for the duration of probiotic therapy, they indicate that *L. plantarum* is capable of inducing large scale compositional changes in the intestinal flora.

## Modulate Human Immune Function

Maintenance of intestinal immune homeostasis is strongly affected by the intestinal microbiota and its interaction with the mucosa. This influence is likely at least partially mediated through the interaction of bacterial cell-wall components or secreted bacterial products with host epithelial or immune effector cells in the intestinal mucosa (de Vos et al., 2017). The nature of these interactions, and their specific immune outcomes are dependent on the species and strain of the participating commensal bacteria, and even though some bacteria have been proven beneficial in certain contexts they may not be beneficial under all clinical circumstances (Gilliland, 1990; Blum and Schiffrin, 2003). Broadly, *L. plantarum* has been demonstrated to have an anti-inflammatory influence on human mucosal immunity. In line with this observation, administration of *L. plantarum* has been shown to reduce immune-mediated pathology in several experimental models of inflammatory diseases such as atopic dermatitis, colitis, and diabetes (Liu et al., 2011; Han et al., 2012; Jang et al., 2014; Kim I. S. et al., 2019; Toshimitsu et al., 2019).

It is important to note that while the immune-modulatory effect of *L. plantarum* in the context of inflammatory diseases is reasonably well-established, the influence it may have in a sub-clinical inflammatory, or healthy setting may differ significantly. A pair of large-scale *in vitro* screens assessed the capacity of a variety of different *L. plantarum* strains to stimulate cytokine production from either human PBMCs or purified human dendritic cells (Meijerink et al., 2010; van Hemert et al., 2010). Each of these studies assessed the capacity of over 40 different strains of *L. plantarum* to induce IL-12 and IL-10 and compared them to the WCSF-1 reference strain. These studies not only revealed significant inter-strain variability in the capacity to induce either IL-10 or IL-12 but also the ratio at which these cytokines were induced. To identify *L. plantarum* genes with a potential role in modulating human immune responses, inter-strain genetic diversity was correlated with specific capacity to induce cytokines from PBMCs or dendritic cells using comparative genome hybridization. Each of these studies identified a number of candidate genes, however both of them implicated genes involved in the production, transport and regulation of plantaricin compounds as having a significant influence on differential inter-strain cytokine induction capacity. Strains with variations in the plantaricin operon generally induced much higher IL-10/IL-12 ratios and more TNFα than those that did not, potentially making them more desirable probiotics (Meijerink et al., 2010; van Hemert et al., 2010).

**Figure 1:**
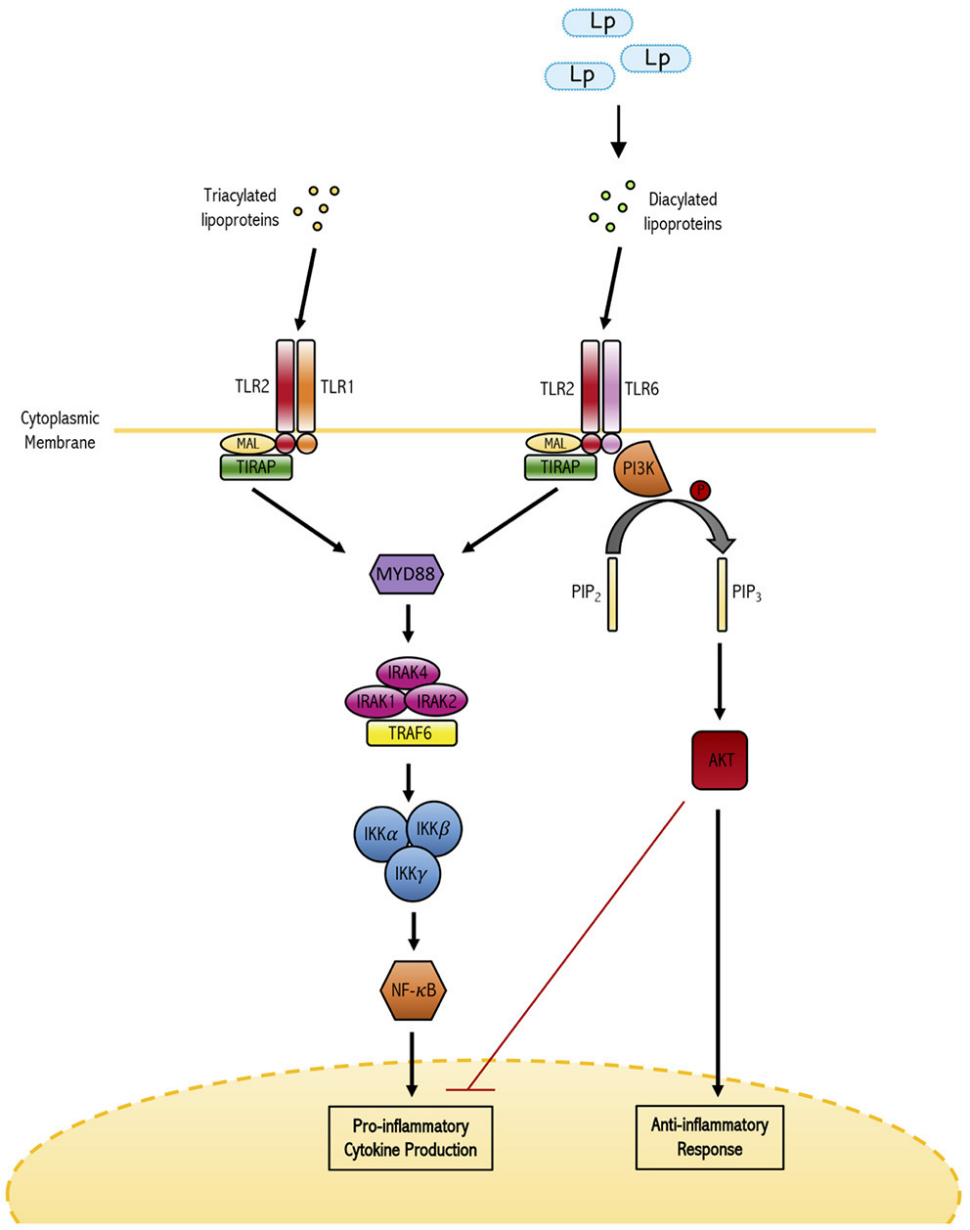
The dual role of TLR-2 in maintaining immune homeostasis. TLR-2 has the capacity to form heterodimers with either TLR-1 or TLR-6. TLRl/2 heterodimers have been shown to bind triacylated lipoproteins frequently derived from gram-negative bacterial species and induce the production of pro-inflammatory cytokines. On the other hand, the TLR2/6 heterodimer binds diacylated lipoproteins derived from gram-positive bacteria (such as *L. plantarum*) and induces anti-inflammatory responses. In this way, TLR-2 signaling plays a major role in differential responses to commensal and potentially pathogenic bacteria.

On the immune sensory side of the interaction, recognition of *L. plantarum* and other lactic acid probiotics by the mucosal immune system is required for their capacity to confer regulatory benefits to the host. Glycolipids derived from *L. plantarum* have been shown to bind to and signal through the glycolipid pattern recognition receptor Mincle modulating host immune status (Shah et al., 2016). Toll-like receptors (TLRs) have a crucial role in innate defense against invading pathogens and serve as a first-line sensory arm of the immune system. Ren and colleagues demonstrated that TLR engagement and downstream signaling, particularly that of the TLR2/TLR6 heterodimer, is an essential element of the immunomodulatory capacity of *L. plantarum* (Ren et al., 2016) (Figure 1). Blocking TLR signaling or inhibiting specific formation of the TLR2/TLR6 heterodimer resulted in significant reduction in *L. plantarum* induced activation of NF-κB/AP-1 signaling, as well as IL-6 and IL-10 production from THP-1 derived macrophages. This is an interesting finding because of the pivotal nature of TLR-2 signaling in maintaining immune homeostasis. This balancing function of TLR-2 is related to its capacity to form heterodimers with both TLR-1 and TLR-6. The TLR1/TLR2 heterodimer has been shown to induce pro-inflammatory cytokines like IL-12 and IL-17, while the TLR2/TLR6 heterodimer has been shown to induce tolerogenic IL-10 based responses (Depaolo et al., 2008; Morita et al., 2017). Fittingly, TLR2/TLR6 signaling agonists have been utilized in the treatment or prevention of many inflammatory diseases such as IBD and mucositis (Depaolo et al., 2008; Sultani et al., 2012). Specifically, TLR2/TLR6 heterodimers promote regulatory T (Treg) cell responses (Ren et al., 2016). Perhaps in further support of the previously described findings, multiple *in vivo* murine studies have demonstrated that administration of *L. plantarum* increased the frequency of both Tregs and regulatory CD103+ dendritic cells. Smelt and colleagues demonstrated administration of *L. plantarum* WCFS1 via intra-gastric gavage every day for 5 days to healthy Balb/c mice induced an increase in the frequency of CD103+ dendritic cells and Tregs in the spleen of treated mice relative to controls (Smelt et al., 2012). In addition, *L. plantarum* administration reduced specific splenic Th2 cell cytokine responses after *ex vivo* re-stimulation.

This result was confirmed and expanded upon in a subsequent study which also observed that administration of *L. plantarum* WCSF1 to healthy mice increased splenic infiltration of regulatory dendritic cells and Tregs and attenuated T helper 2 type responses (Bermudez-Brito et al., 2018). This study expanded upon the previous report by demonstrating that host-microbe interaction at the Peyer's patches was enough to induce immunomodulation of host DCs and T cells. This is of interest as probiotics are hypothesized to modulate the immune system through two different pathways: (i) probiotics may be sampled by M cells in the Peyer's patches and passaged to modulate immune effector cells beneath the epithelium or (ii) specific intestinal DCs in the mucosal lamina propria sense probiotics via pattern-recognition receptors. This contact with DCs, via either pathway, influences the maturation of APCs and impacts their interaction with other immune effectors cells, thereby determining the polarization state of the subsequent immune response (Hardy et al., 2013; Bermudez-Brito et al., 2018). Using bioluminescently labeled *L. plantarum*, Bermudez-Brito and colleagues demonstrate that in healthy mice, M-cell sampling of Peyer's patches was a relatively rare occurrence, and that recognition of *L. plantarum* by resident dendritic cells in the Peyer's patches or isolated lymphoid follicles was sufficient to modulate systemic immune responsiveness.

Evidence of *L. plantarum* induced immune modulation in healthy humans remains limited but the existing studies lend further support to *in vitro* and murine model studies that suggest *L. plantarum* can skew toward a regulatory immune phenotype. In a recent study, administration of *L. plantarum* strains was found to prevent non-steroidal anti-inflammatory drug induced reductions in circulating Treg populations (de Vos et al., 2017). This study also assessed the impact of *L. plantarum* on memory responses against tetanus toxoid (TT) antigen and found that by inducing the expression of genes associated with the maintenance of T and B cell function and antigen presentation, *L. plantarum* enhanced responses against TT-antigen in T cell polarization studies. Finally, in the complete absence of any disease or inflammatory stress, a double-blind placebo controlled randomized clinical trial demonstrated that administration of *L. plantarum* significantly altered gene expression profiles in the intestinal mucosa of healthy adults. As determined by biopsies taken from the intestinal duodenal mucosa, living but not heat-killed *L. plantarum* significantly modulated NF-κB dependent pathways (van Baarlen et al., 2009). Strikingly, after consumption of *L. plantarum*, significant induction of genes associated with anti-inflammatory activities such as BCL3, ADM, and IκB was observed, potentially correlating with the establishment of a regulatory or tolerance skewed immune environment (van Baarlen et al., 2009). While administration of *L. plantarum* in different growth phases may have differential effects on the host, collectively, these studies demonstrate the profound capacity of *L. plantarum to* modulate the host immune activity, presumably by skewing toward an anti-inflammatory or regulatory state.

## Evidence of Capacity to Treat or Improve Human Disease

There is mounting evidence demonstrating that administration of *L. plantarum* can have a beneficial impact on human health, particularly in the context of managing or preventing inflammatory diseases. In a prospective single-arm open trial, Toshimitsu and colleagues assessed the impact of a 12-week treatment with heat-killed *L. plantarum* OLL2712 on glucose metabolism and chronic inflammation in pre-diabetic individuals (Toshimitsu et al., 2019). This study demonstrated that, compared to baseline values, treatment with *L. plantarum* significantly reduced fasting plasma glucose and serum glycoalbumin and resulted in a significant improvement in insulin resistance (HOMA-IR) and insulin sensitivity (QUICKI) indexes. Chronic inflammation is considered to be a primary cause of metabolic disorders and has previously been shown to cause insulin resistance and impair glucose and lipid metabolism (Fantuzzi et al., 2005). In addition to markers of glucose metabolism the above mentioned study also assessed how *L. plantarum* treatment affected the pro-inflammatory cytokine levels in patient serum. Relative to baseline measurement administration of *L. plantarum* significantly reduced serum MCP-1 and IL-6 levels (Toshimitsu et al., 2019). Stratification revealed that the observed reduction in fasting plasma glucose levels was most prominent in the participants that exhibited high baseline pro-inflammatory cytokine expression. In these participants, *L. plantarum* induced a significant reduction in fasting plasma glucose and improvement in insulin resistance indexes that was not observed in individuals with lower baseline pro-inflammatory cytokine expression.

There is also evidence that *L. plantarum* may have cholesterol lowering effects in hypercholesterolaemic adults, potentially lowering the incidence of coronary heart disease (CHD). In a double-blind randomized trial, Costabile and colleagues assessed the impact of twice-daily administered *L. plantarum* ECGC 13110402 for 12 weeks on several measures of blood lipid content (Costabile et al., 2017). This study demonstrated that administration of *L. plantarum* ECGC 13110402, selected primarily for its particularly high bile-salt hydrolase activity, resulted in statistically significant reductions in low density lipoproteins, total cholesterol, and triacylglycerides, while increasing high density lipoprotein levels. Interestingly, in the over 60-year-old population, *L. plantarum* also induced a significant reduction in systolic blood pressure (Costabile et al., 2017). As no significant side-effects or changes to gastrointestinal function were observed, this study suggests that supplementation of existing treatment strategies with administration of *L. plantarum* ECGC 13110402 may be used to reduce the risk of cardiovascular disease, particularly in high-risk subjects with hypercholesterolaemia.

The beneficial effect of *L. plantarum* in animal models of IBS and IBD is well-established (Liu et al., 2011; Jang et al., 2014; Chen et al., 2017). However, evidence of a similar benefit in humans remains relatively limited. In a randomized double-blind placebo-controlled study, the effect of 4-week treatment with *L. plantarum* 299v was assessed in irritable bowel syndrome patients fulfilling the Rome III diagnostic criteria. Ducrotté and his team observed that treatment with *L. plantarum* 299v was associated with significant reduction in patient pain severity and bloating (Ducrotte et al., 2012). In line with many previous IBS trials, a modest placebo response was observed. Despite this however, 78% of the patients on the *L. plantarum* arm scored the treatment efficacy as excellent or good vs. only 8% of patients on the placebo arm. This study supports the findings of a previous randomized double-blind study that also showed significant pain relief and amelioration of accompanying IBS symptoms in patients receiving 4-week *L. plantarum* 299v treatment in a 20-patient cohort (Niedzielin et al., 2001).

In a landmark study involving more than 4,500 full-term newborns in rural India, Panigrahi and colleagues assessed the efficacy of an orally administered synbiotic containing *L. plantarum* (ATCC-202195) and fructooligosaccharide in preventing infant sepsis. Strain selection and administration protocols for this trial were informed by two pilot studies previously conducted by the same group. The first of these pilot studies demonstrated that *Lactobacillus GG* was a poor colonizer in infants, especially those weighing <1,500 grams (Agarwal et al., 2003). Conversely, the authors subsequently demonstrated that a synbiotic preparation of *L. plantarum* and fructooligosaccharide persisted in infants for months after therapy ended (Panigrahi et al., 2008). In this pilot study, after 7 days of synbiotic treatment, initiated between 1 and 3 days after birth, *L. plantarum* was cultured from 84% of the treated infants after 3 days of treatment and from 95% of the infants on day 28 after birth (Panigrahi et al., 2008). Of the infants colonized at day 28, 100, 94, 88, 56, and 32% remained colonized at months 2, 3, 4, 5, and 6 suggesting that, unlike *Lactobacillus GG, L. plantarum* is capable of efficiently and durably colonizing the newborn gut.

Consequently, Panigrahi and colleagues designed their trial to assess the efficacy of *L. plantarum* and fructooligosaccharide administered to newborns for 7 days beginning on day 2–4 of life in preventing the primary outcome of neonatal sepsis and death (Panigrahi et al., 2017). Community volunteers identified 7,089 cases of suspect sepsis during a population-based screening of women delivering in the study area, of which 4,556 newborns were identified to have sign/symptoms of sepsis by trained pediatricians. They were randomized to receive either the synbiotic preparation (*n* = 2,278) or the placebo (*n* = 2,278), and treated after collection of blood for Bactec culture. A significant (42%) reduction in the primary outcome of neonatal sepsis and death was observed, from 9% in the placebo arm to 5.4% in the treatment arm, indicating that only 27 infants would need to be treated to prevent one primary outcome (Panigrahi 2017). Importantly, a significant reduction was observed in culture positive septicaemia with 27 cases in the control group and only 6 in the treatment group (risk ratio (RR) = 0.22). Synbiotic treatment was also associated with a significant reduction in lower respiratory tract infections (34%) potentially suggesting that probiotic administration not only enhanced gastrointestinal mucosa but also systemic immunity (Panigrahi et al., 2017). Of the evaluated secondary outcomes synbiotic treatment was also associated with a significant reduction in local infections and a greater increase in weight gained since birth at day 60 of life. The observed 42% reduction in sepsis was significantly greater than the anticipated reduction and facilitated early termination of the trial. The results of this trial stand in contrast to two other studies that used probiotics to target sepsis in very preterm infants (Jacobs et al., 2013; Costeloe et al., 2016) and a third, smaller trial, conducted in low birth weight infants in India (Sinha et al., 2015). These differences may be related to probiotic choice, administration schedule or the addition of a prebiotic supplement, but suggest that administration of *L. plantarum* may be capable of reducing the risk of sepsis/pSBI and lower respiratory infections for neonates in developing countries.

## Future Directions

*L. plantarum* is capable of inhabiting a wide variety of ecological niches including the human gastrointestinal mucosa. Due to their unique evolutionary history, strains of this species possess a wide range of probiotic characteristics and have shown promise in numerous experimental disease models, as well as human trials. Increasing our understanding of strain specific variation, particularly in relation to probiotic characteristics and imparted health benefits, is now paramount. Characterizing *L. plantarum* strains in such a manner will facilitate standardization of research applications and allow for the more rapid and efficient validation of specific strains for defined health applications. Beyond *L. plantarum*, developing a more comprehensive understanding of the specific genetic and phenotypic characteristics that impart specific probiotic functionalities or health benefits may provide the key for identifying or perhaps engineering more effective probiotic strains. The inherent genetic flexibility that facilitates the nomadic lifestyle of *L. planatrum* may render it particularly amenable to such engineering and may facilitate the production of highly effective probiotic strains.

## Author Contributions

MF wrote the review with TK. PP provided significant revisions. All authors contributed to the article and approved the submitted version.

## Conflict of Interest

PP isolated and further developed the strain ATCC 202195 that has been licensed to Chr Hansen for commercial production and use in food/supplement. The remaining authors declare that the research was conducted in the absence of any commercial or financial relationships that could be construed as a potential conflict of interest.

## Publisher's Note

All claims expressed in this article are solely those of the authors and do not necessarily represent those of their affiliated organizations, or those of the publisher, the editors and the reviewers. Any product that may be evaluated in this article, or claim that may be made by its manufacturer, is not guaranteed or endorsed by the publisher.
